# Nonalcoholic fatty liver disease is an independent risk factor for ischemic stroke after revascularization in patients with Moyamoya disease: a prospective cohort study

**DOI:** 10.1186/s12944-024-02065-5

**Published:** 2024-03-17

**Authors:** Bojian Zhang, Junsheng Li, Chaofan Zeng, Chuming Tao, Qiheng He, Chenglong Liu, Zhiyao Zheng, Zhikang Zhao, Siqi Mou, Wei Sun, Jia Wang, Qian Zhang, Rong Wang, Yan Zhang, Peicong Ge, Dong Zhang

**Affiliations:** 1 Department of Neurosurgery, Beijing Tiantan Hospital, Capital Medical University, Beijing, 100070 China; 2 China National Clinical Research Center for Neurological Diseases, Beijing, China; 3 Center of Stroke, Beijing Institute for Brain Disorders, Beijing, China; 4 Beijing Key Laboratory of Translational Medicine for Cerebrovascular Disease, Beijing, China; 5 Beijing Translational Engineering Center for 3D Printer in Clinical Neuroscience, Beijing, China; 6 Department of Neurosurgery, Beijing Hospital, National Center of Gerontology, Beijing, 100730 China; 7 Institute of Geriatric Medicine, Chinese Academy of Medical Sciences, Beijing, China

**Keywords:** Moyamoya disease, Stroke, Risk factor, Prognosis, NAFLD

## Abstract

**Background:**

The study aimed to investigate the association between nonalcoholic fatty liver disease (NAFLD) and ischemic stroke events after revascularization in patients with Moyamoya disease (MMD).

**Methods:**

This study prospectively enrolled 275 MMD patients from September 2020 to December 2021. Patients with alcoholism and other liver diseases were excluded. NAFLD was confirmed by CT imaging or abdominal ultrasonography. Stroke events and modified Rankin Scale (mRS) scores at the latest follow-up were compared between the two groups.

**Results:**

A total of 275 patients were enrolled in the study, among which 65 were diagnosed with NAFLD. Univariate logistic regression analysis showed that NAFLD (*P* = 0.029) was related to stroke events. Multivariate logistic regression analysis showed that NAFLD is a predictor of postoperative stroke in MMD patients (OR = 27.145, 95% CI = 2.031–362.81, *P* = 0.013). Kaplan-Meier analysis showed that compared with MMD patients with NAFLD, patients in the control group had a longer stroke-free time (*P* = 0.004). Univariate Cox analysis showed that NAFLD (*P* = 0.016) was associated with ischemic stroke during follow-up in patients with MMD. Multivariate Cox analysis showed that NAFLD was an independent risk factor for stroke in patients with MMD (HR = 10.815, 95% CI = 1.259–92.881, *P* = 0.030). Furthermore, fewer patients in the NAFLD group had good neurologic status (mRS score ≤ 2) than the control group (*P* = 0.005).

**Conclusion:**

NAFLD was an independent risk factor for stroke in patients with MMD after revascularization and worse neurological function outcomes.

## Background

Moyamoya disease (MMD) is an infrequent cerebrovascular disorder characterized by the gradual constriction of terminal sections of the internal carotid arteries, accompanied by the emergence of an anomalous vascular network at the base of the brain. MMD can result in either ischaemic or haemorrhagic strokes, although ischemic stroke symptoms are more commonly observed [1–3]. MMD significantly contributes to stroke incidence, primarily in East Asian populations [4–6]. Its age distribution shows two distinct peaks, occurring at approximately 5 years old and 40 years old [7, 8]. No effective drug treatment for MMD has yet been discovered.

Surgical treatment is recognized for its ability to significantly improve intracranial blood supply and alleviate ischemic symptoms, although it cannot halt the progression of the disease [9]. Previous studies have shown considerable interindividual variability in the prognosis of MMD patients after revascularization, and the probability of recurrence of ischaemic stroke after surgery ranges from 5–24.4% [10–12], leading to severe neurological dysfunction. Stroke patients require long-term medical care and rehabilitation, and the burden of postoperative stroke on families and society cannot be ignored. According to the previous study, lower high-density lipoprotein cholesterol (HDL-C) and hypertension increase the probability of ischaemic stroke in MMD patients [13, 14]. However, research on risk factors for stroke after surgical treatment remains limited. Further investigation is required to explore the causes of stroke in patients with MMD during long-term follow-up.

Non alcoholic fatty liver disease (NAFLD) is a prevalent chronic liver condition marked by the accumulation of excess fat in the liver, and it is unrelated to alcohol consumption [15]. Although its precise cause remains elusive, it is associated with various factors, including diet, lifestyle, genetics, and metabolic abnormalities. Factors such as obesity, a high-fat diet, physical inactivity, and genetic predisposition can all contribute to the development of NAFLD. Previous studies have unequivocally established NAFLD as a cardiovascular and cerebrovascular disease risk factor [16, 17]. Recently, NAFLD severity has been recognized to be a significant factor in the risk factor for ischaemic stroke [18]. It has been confirmed in some studies that the anomalies in indicators such as γ-glutamyltransferase (GGT), TG, and HDL-C caused by NAFLD are related to ischemic stroke [19, 20]. The development of ischaemic cardiovascular and cerebrovascular diseases is influenced by NAFLD. In summary, NAFLD has the potential to adversely affect the incidence of ischaemic stroke. However, for MMD patients with ischaemic stroke as the main symptom, the specific association between ischaemic stroke and NAFLD has not yet been studied. Therefore, the study explored the relationship between NAFLD and postoperative ischemic strokes in patients with MMD.

## Methods

### Patient date and study design

**Fig. 1:**
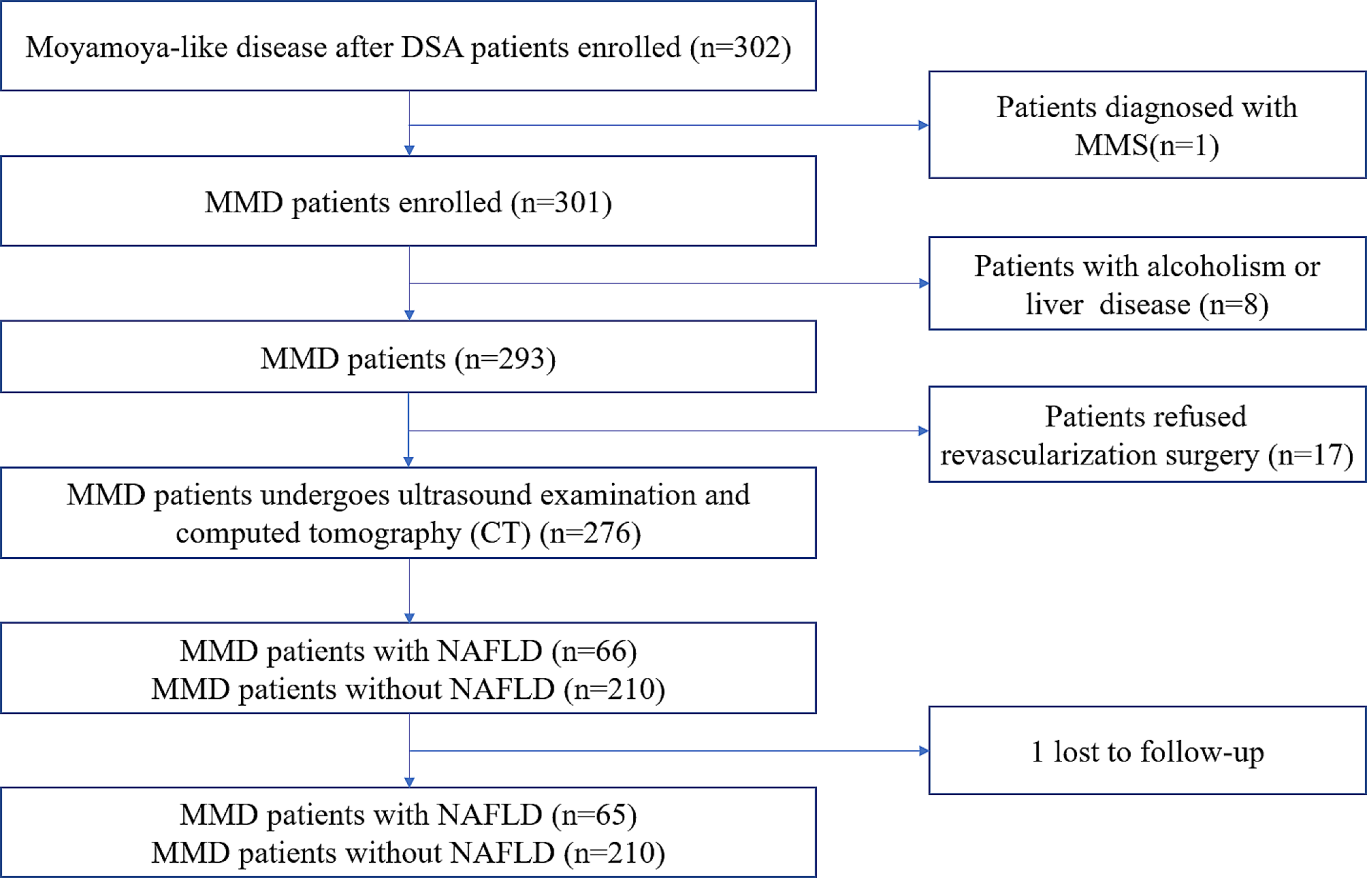
Flowchart of the study. NAFLD, non-alcoholic fatty liver disease. MMD, Moyamoya disease. MMS, Moyamoya syndrome.

Three hundred and two patients who were diagnosed with moyamoya-like disease were consistently included in the study following digital subtraction angiography (DSA) from September 2020 to December 2021. Patients diagnosed with moyamoya syndrome (MMS) due to known causes, such as isolated occlusion of the cervical internal carotid artery, were not included in the investigation. Patients with a history of excessive alcohol consumption or concurrent liver conditions (such as hepatic cysts and viral hepatitis, etc.) were also excluded. All patients enrolled in this study were diagnosed according to the 2012 Japanese MMD guideline [21]. Patients who refused revascularization surgery were not included. All patients were monitored through either clinical appointments or phone conversations. Patients lost to follow-up were also excluded from this study. Of the initial 302 patients with MMD, 8 were excluded due to alcoholism (defined as more than 40 g per day or 280 g per week for women, and 60 g per day or 420 g per week for men) [22], 17 received conservative treatment, 1 was diagnosed with MMS, and 1 was lost to follow-up. Ultimately, 275 MMD patients entered the final study phase, with 257 patients identified based on the liver and spleen CT value ratio and 18 patients diagnosed via ultrasound (Fig. 1).

### Surgical modalities

In this study, patients underwent one of three surgical methods: direct bypass, indirect bypass, or a combined bypass procedure. Direct bypass involves the end-to-side anastomosis between the superficial temporal artery and the cortical branch of the middle cerebral artery (M4 segment), referred to as STA-MCA. Indirect bypass includes procedures such as encephalo-duro-arterio-synangiosis connection or encephalo-duro-arterio-muscle-synangiosis connection, also known as EDAS or EDAMS. Combined bypass refers to a combination of the above two techniques.

### Diagnosis of NAFLD

The primary diagnostic criteria for the diagnosis of the NAFLD were based on ultrasound or computed tomography (CT). The key features of ultrasound diagnosis encompass the following points: (1) compared to the renal or splenic region, there was a notable increase in near-field echogenicity within the liver region, which was accompanied by a gradual attenuation of the far-field echo and (2) mild enlargement of the liver with an indistinct intrahepatic cavity structure. CT scans reveal a diffuse decrease in liver density, with the liver-spleen CT value ratio ≤ 1 [23]. 

### Follow-Up and outcomes

Ischaemic strokes were regarded as the primary outcome measure, defined as cerebral infarctions accompanied by neurological deficits lasting more than 24 h and confirmed to be related to the infarctions observed on MRI or CT scans. Additionally, the modified Rankin Scale (mRS) score was documented to evaluate the neurological status of every patient., categorized into two levels: good (mRS score ≤ 2) or poor (mRS score > 2).

### Statistical analysis

The study performed all statistical analyses using IBM SPSS Statistics software (version 26.0; IBM Corp.). Categorical variables are presented as numbers and percentages. The median and interquartile range were used to describe continuous variables with skewed distributions. Continuous variables with a normal distribution are described using the mean and standard deviation. Continuous variables were analysed using the Kruskal-Wallis test, Mann-Whitney U test, and t-test, while categorical variables were assessed with the chi-square test. To analyse the association between quartiles of the liver-to-spleen CT ratios and clinical baseline characteristics, the study used the Spearman correlation test. Risk factors for stroke events were identified through both univariate and multivariate logistic regression analyses. Stroke-free survival was analysed using Kaplan-Meier curves and the log-rank test. Finally, the study examined the relationship between NAFLD and the outcome using both univariate and multivariate Cox proportional hazard models. The result was deemed statistically significant when a two-sided P-value was less than 0.05.

## Results

### Baseline characteristics

Sixty-five MMD patients were identified to have NAFLD. MMD patients with NAFLD exhibited elevated levels of several biomarkers, including triglyceride (TG), gamma-glutamyl transpeptidase (GGT), uric acid (UA), BMI, aspartate transaminase (AST), and alanine transaminase (ALT), whereas reduced levels of HDL-C. The above statistical results all had *P* values < 0.05. Furthermore, a significant sex difference was confirmed when compared to MMD patients without NAFLD (*P* = 0.028). Age, hypertension status, diabetes status, hyperlipidemia status, Suzuki stage, surgical option, and admission mRS score were also compared, However, no statistical difference were not found, as shown in Table 1.

**Table 1:** Comparison of baseline characteristics between NAFLD group and control group

Variable	Control	NAFLD	P value
n (%)	210	65	-
Age, y, mean ± SD	35.62(± 16.217)	38.00(± 13.959)	0.448
Sex(male), n (%)	90(42.9)	38(58.5)	0.028*
Clinical features, mean ± SD			
Heart rate, bpm	78.63(± 6.872)	78.62(± 6.232)	0.912
SBP, mmHg	129.57(± 14.914)	134.17(± 17.335)	0.146
DBP, mmHg	81.19(± 10.586)	82.78(± 11.013)	0.716
BMI, kg/m2	23.85(± 4.551)	27.07(± 4.647)	< 0.001*
History of risk factor, n (%)			
Hypertension	69(32.9)	23(35.4)	0.706
Diabetes	28 (13.3)	11(18.5)	0.305
Hypercholesterolemia	27(12.9)	9(13.8)	0.836
Smoking	31(14.8)	15(23.1)	0.116
Laboratory results, median (IQR)			
WBC count, 10^9^/L	7.04(2.27)	6.61(3.52)	1.000
LY count, 10^9^/L	1.91(0.92)	1.89(0.91)	0.851
PLT count, 10^9^/L	256.00(84)	256(56)	0.672
HDL-C, mmol/L	1.30(0.40)	1.14(0.33)	0.002*
LDL-C, mmol/L	2.185(1.20)	2.20(1.55)	0.871
Hcy µmol/L	12.10(6.42)	12.87(6.57)	0.308
ALT, U/L	16.95(15.3)	25.4(27.9)	< 0.001*
AST, U/L	17.00(6.2)	20.00(13.9)	0.003*
GGT, mmol/L	19.00(16.0)	27.50(20.7)	< 0.001*
Cr, µmol/L	52.85(20.9)	53.70(24.5)	0.180
UA, µmol/L	294.95(108.9)	351.20(117.5)	< 0.001*
TG, mmol/L	1.055(0.71)	1.53(1.21)	< 0.001*
TC, mmol/L	3.93(1.38)	4.21(1.53)	0.815
ApoA, g/L	1.295(0.31)	1.21(0.32)	0.087
ApoB, g/L	0.78(0.26)	0.82(0.34)	0.228
Primary symptom n (%)			0.707
Infarction	83(39.5)	24(36.9)	
Non-infarction	127(60.5)	41(63.1)	
Suzuki stage, n (%)			0.425
1–3	151(71.9)	50(76.9)	
4–6	59(28.1)	15(23.1)	
Surgical option, n (%)			0.950
Direct bypass	96(45.7)	30(46.2)	
Indirect bypass	114(54.3)	35(53.8)	
Admission mRS, n (%)			0.858
0–2	189(90)	58(89.8)	
3–5	21(10)	7(10.2)	

SBP, systolic blood pressure; DBP, diastolic blood pressure; BMI, body mass index; WBC, white blood cell; LY, lymphocyte; PLT, platelet; HDL-C, high-density lipoprotein cholesterol; LDL-C, low-density lipoprotein cholesterol; Hcy, homocysteine; ALT, alanine transaminase; AST, aspartate transaminase; GGT, γ-glutamyltransferase; Cr, creatinine; UA, uric acid; TG, triglyceride; TC, total cholesterol; ApoA, apolipoprotein A; ApoB, apolipoprotein B; SD, standard deviation; IQR, interquartile range. Suzuki staging and posterior circulation involvement were defined on the operative side. *P < 0.05, significant difference

The MMD patients diagnosed with NAFLD based on the CT ratio of the liver and spleen were divided into four groups based on the CT ratio, and baseline differences and correlations between the groups were analysed. The distribution differences of gender, hypertension, and diabetes between each group were statistically significant. In addition, statistical differences were identified among the four groups in TG, GGT, ALT, AST, and UA, and they were significantly negatively correlated with the liver and spleen CT ratio. Significant differences were identified among HDL-C groups, with a significant positive correlation observed with the liver-to-spleen CT ratio, as presented in Table 2.

**Table 2:** Baseline Characteristics of MMD patients according to the quartiles of CT (liver/spleen)

Variables	CT (liver/spleen)				P-Value	Spearman Correlation	P -Value
	Q1 (0.62–1.06, *n* = 64)	Q2 (1.06–1.21, *n* = 64)	Q3 (1.21–1.30, *n* = 65)	Q4 (1.30–1.87 *n* = 64)			
Age, y, mean ± SD	38.14 ± 14.36	39.36 ± 14.36	32.32 ± 17.02	34.17 ± 17.03	0.043	-0.118	0.059
Sex(male), n (%)	37(57.8)	36(56.3)	26(40)	17(26.6)	0.001*	-0.247	< 0.001*
Clinical features, mean ± SD							
Heart rate, bpm	79.42 ± 6.125	78.16 ± 7.44	79.48 ± 7.53	78.47 ± 5.75	0.611	-0.051	0.416
SBP, mmHg	134.02 ± 17.23	133.84 ± 14.14	127.63 ± 13.57	125.61 ± 15.47	0.002*	-0.186	0.003*
DBP, mmHg	82.41 ± 11.39	82.39 ± 9.536	80.58 ± 9.04	79.00 ± 11.41	0.232	-0.067	0.281
BMI, kg/m2	26.91 ± 5.10	25.34 ± 4.81	23.12 ± 4.42	22.80 ± 3.93	< 0.001*	-0.314	< 0.001*
History of risk factor, n (%)							
Hypertension	24(37.5)	28(43.8)	16(24.6)	15(23.4)	0.034	-0.147	0.019*
Diabetes	12(18.8)	15(23.4)	6(9.2)	3(4.7)	0.008*	-0.182	0.003*
Hypercholesterolemia	11(17.2)	9(14.1)	3(4.6)	7(10.9)	0.144	-0.098	0.116
Smoking	10(15.6)	15(23.4)	5(7.7)	8(12.5)	0.082	-0.079	0.204
Laboratory results, median (IQR)							
HDL-C, mmol/L	1.14(0.34)	1.25(0.45)	1.34(0.35)	1.35(0.44)	0.007*	0.211	0.001*
LDL-C, mmol/L	2.385(1.52)	2.245 (1.31)	2.07(0.88)	2.205(1.11)	0.543	-0.025	0.691
TG, mmol/L	1.51(1.33)	1.08(0.93)	0.89 (0.73)	1.07(0.58)	< 0.001*	-0.237	< 0.001*
TC, mmol/L	4.34(1.52)	3.97(1.46)	3.76(0.96)	3.99(1.39)	0.614	-0.031	0.618
GGT, mmol/L	27.15(21.2)	24.1(22.1)	16.8(14.8)	15.5(8.4)	< 0.001*	-0.365	< 0.001*
ALT, U/L	25(26.2)	20.5(0.34)	14.5(11.8)	14.55(11.9)	0.001*	-0.339	< 0.001*
AST, U/L	19.75(12.4)	16.7(4.6)	17(7.3)	18(8.2)	0.033*	0.146	0.019*
APO-A1, g/L	1.21(0.33)	1.30(0.36)	1.30(0.29)	1.30 (0.38)	0.381	0.117	0.062
APO-B, g/L	0.815(0.368)	0.86(0.328)	0.74(0.235)	0.785(0.24)	0.130	-0.106	0.090
HCY, µmol/L	12.94(6.87)	12.985(6.28)	11.23(4.75)	12.115(6.37)	0.715	-0.072	0.250
Cr, µmol/L	54.55(24.7)	54.15(25.1)	51.60(19.8)	50.60(19.4)	0.231	-0.124	0.047*
UA, µmol/L	350.1(121.6)	316.45(118)	280.1(93.1)	294.95(95.2)	< 0.001*	-0.296	< 0.001*
WBC count, 10^9^/L	7.385(3.57)	7.1(1.96)	6.93(2.28)	6.75(2.84)	0.519	-0.056	0.373
LY count, 10^9^/L	2.02(1.06)	2.01(0.82)	1.89(1.11)	1.81(0.97)	0.830	-0.075	0.228
PLT count, 10^9^/L	256.5(59)	266(95)	256(82)	254.5(96)	0.279	-0.035	0.579
Primary symptom n (%)					0.079	-0.091	0.145
Infarction	24(37.5)	31(48.4)	27(41.5)	17(24.7)			
Non-infarction	40(62.5)	33(51.6)	38(58.5)	47(73.4)			
Suzuki stage, n (%)					0.326	0.086	0.170
1–3	51(79.7)	49(76.6)	43(66.2)	46(71.9)			
4–6	13(20.3)	15(23.4)	22(33.8)	18(28.1)			

* *P* < 0.05, significant difference

### Surgical complications

Sixteen patients (24.6%) in the NAFLD group experienced surgical complications, whereas 37 patients (17.6%) in the control group experienced postoperative complications. However, the statistical difference was not observe in the occurrence of surgical complications (*P* = 0.211).

Regarding specific outcomes, 7 patients (10.8%) in the NAFLD group experienced infarctions, while 16 patients (7.6%) in the control group experienced infarctions. Additionally, 5 patients (7.7%) in the NAFLD group had transient ischemic attacks (TIAs), while 13 patients (6.2%) in the control group experienced TIAs. Two patients (3.1%) in the NAFLD group were diagnosed with hyperperfusion syndrome (confirmed by patient symptoms and CT perfusion), whereas 6 patients (2.9%) in the control group exhibited hyperperfusion syndrome. 1 patient (1.5%) in the NAFLD group developed symptomatic epilepsy, whereas none of the control group experienced this complication. Additionally, one patient (1.5%) in the control group had intracranial hemorrhage, while no patient in the NAFLD group had this complication. Finally, one patient in each group developed postoperative infection. Statistically, there is no difference for the above.

### Clinical outcomes of NAFLD

During the follow-up period, 7 patients with cerebral infarction were observed. The median observation time in the NAFLD group was 19 months, while in the control group, it was 23 months, and significantly longer than the former (*P* = 0.031).

Stroke events were observed in 4 MMD patients with NAFLD, while there were 2 people in the control group who had stroke events. Univariate logistic regression analysis revealed that diabetes, hypercholesterolemia, white blood cell (WBC) count, and NAFLD (*P* = 0.029) were associated with stroke events. According to the multivariate logistic regression analysis, NAFLD emerged as a predictor of postoperative recurrence of stroke in MMD patients (OR = 27.145, 95% CI = 2.031–362.81, *P* = 0.013, as shown in Table 3).

**Table 3:** Logistic regression analyses for ischemic stroke

Variables	Univariate analyses		Multivariate analyses	
	OR (95%CI)	P Value	OR (95%CI)	P Value
Age	1.046 (0.979–1.117)	0.186		
Sex	5.935 (0.684–51.483)	0.106		
BMI	1.099 (0.950–1.272)	0.205	0.816 (0.606–1.099)	0.180
Smoking	2.557 (0.454–14.391)	0.287		
Hypertension	4.114 (0.739–22.890)	0.106	2.268 (0.152–33.848)	0.553
Hypercholesterolemia	7.152 (1.386–36.913)	0.019*	69.265(2.26-2121.62)	0.015*
Diabetes	33.429 (3.793-294.603)	0.002*	119.67(5.15-7746.41)	0.005*
Primary symptom (%)				
Infarction	1.587 (0.314–8.009)	0.576		
Non-infarction	Reference	-		
Laboratory results				
WBC count, 10^9^/L	1.368 (1.090–1.718)	0.007*		
LY count, 10^9^/L	0.911 (0.283–2.938)	0.877		
PLT count, 10^9^/L	1.007 (0.998–1.017)	0.139		
Cr, µmol/L	1.027 (0.991–1.065)	0.146		
UA, µmol/L	0.999 (0.990–1.008)	0.787		
ALT, U/L	1.014 (0.989–1.039)	0.278		
HDL, mmol/L	0.175 (0.009–3.528)	0.255	1.232 (0.051–29.524)	0.897
LDL, mmol/L	0.673 (0.234–1.931)	0.461		
TG, mmol/L	1.295 (0.681–2.461)	0.431		
TC, mmol/L	0.609 (0.260–1.428)	0.254		
GGT, mmol/L	0.996 (0.956–1.037)	0.832	0.921 (0.823–1.031)	0.153
HCY, µmol/L	1.047 (0.968–1.132)	0.251		
ApoA, g/L	0.910 (0.044–18.830)	0.952		
ApoB, g/L	0.652 (0.016–26.758)	0.822		
Suzuki stage, n (%)				
1–3	Reference	-		
4–6	1.368 (0.245–7.630)	0.721		
Surgical option (%)				
Indirect bypass	Reference	-	Reference	-
Direct bypass	0.585 (0.105–3.246)	0.539	0.065 (0.003–1.291)	0.073
NAFLD	6.820 (1.220–38.130)	0.029*	27.145 (2.031–362.81)	0.013*

OR, odds ratio; CI, confidence interval; * *P* < 0.05, significant difference

**Fig. 2:**
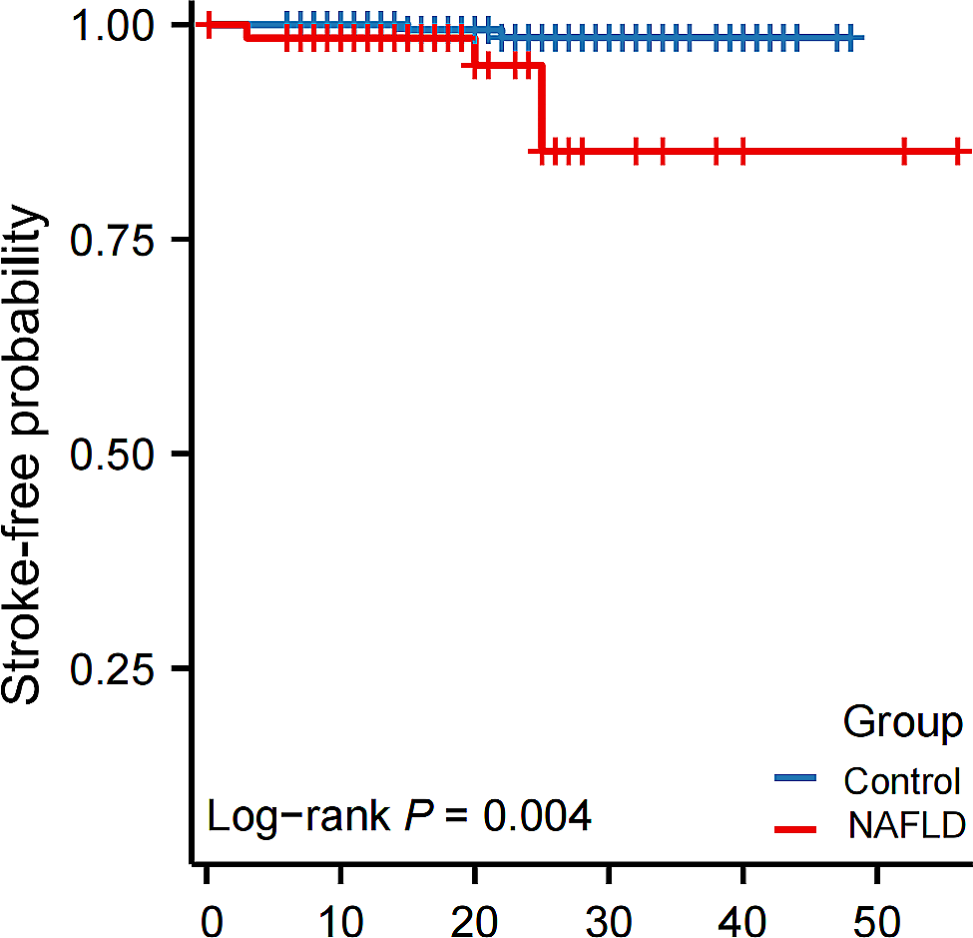
Stroke-free probability. NAFLD, non-alcoholic fatty liver disease

Kaplan-Meier analysis revealed a notable difference in the distribution of stroke-free survival between MMD patients with NAFLD and controls (Fig. 2). Potential risk factors for stroke during follow-up in postoperative MMD patients were analysed using univariate Cox analysis. In MMD patients, hyperlipidemia, diabetes, WBC count, and NAFLD (*P* = 0.016) were associated with ischemic stroke during follow-up (Table 4). After adjusting for all possible covariates, the results revealed that the presence of NAFLD was a risk factor independent of other factor for stroke in patients with postoperative MMD (HR = 10.815, 95% CI = 1.259–92.881, *P* = 0.030, Table 4).

**Table 4:** Cox analyses for recurrent ischemic stroke

Variables	Univariate analyses		Multivariate analyses	
	HR (95%CI)	P Value	HR (95%CI)	P Value
Age	1.044 (0.978–1.114)	0.199		
Sex	5.645 (0.658–48.423)	0.115		
BMI	1.109 (0.959–1.283)	0.163	0.954 (0.747–1.219)	0.708
Smoking	2.390 (0.437–13.068)	0.315		
Hypertension	4.092 (0.749–22.351)	0.104	1.614 (0.209–12.480)	0.647
Hypercholesterolemia	7.349 (1.481–36.472)	0.015*	41.826 (2.188-799.396)	0.013*
Diabetes	25.648 (2.993–219.82)	0.003*	26.938 (1.779–407.820)	0.018*
Primary symptom (%)				
Infarction	1.568 (0.316–7.771)	0.582		
Non-infarction	Reference	-		
Laboratory results				
WBC count, 10^9^/L	1.483 (1.203–1.828)	< 0.05*		
LY count, 10^9^/L	0.863 (0.254–2.928)	0.813		
PLT count, 10^9^/L	1.007 (0.998–1.016)	0.114		
Cr, µmol/L	1.038 (0.995–1.083)	0.085		
UA, µmol/L	0.999 (0.991–1.008)	0.859		
ALT, U/L	1.016 (0.991–1.042)	0.212		
HDL, mmol/L	0.247 (0.015–4.063)	0.328	1.259 (0.080-19.715)	0.870
LDL, mmol/L	0.639 (0.221–1.849)	0.409		
TG, mmol/L	1.505 (0.723–3.133)	0.274		
TC, mmol/L	0.586 (0.234–1.470)	0.255		
GGT, mmol/L	0.996 (0.952–1.041)	0.844	0.930 (0.834–1.036)	0.185
HCY, µmol/L	1.037 (0.958–1.122)	0.370		
ApoA, g/L	0.783 (0.043–14.230)	0.869		
ApoB, g/L	0.42 (0.009–21.294)	0.669		
Suzuki stage, n (%)				
1–3	Reference	-		
4–6	0.994 (0.465–2.124)	0.987		
Surgical option (%)				
Indirect bypass	Reference	-	Reference	-
Direct bypass	0.561 (0.103–3.065)	0.505	0.048 (0.002–1.113)	0.058
NAFLD	8.066 (1.474–44.134)	0.016*	10.815 (1.259–92.881)	0.030*

HR, Hazard Ratio; CI, confidence interval; * *P* < 0.05, significant difference

In terms of neurological status, 55 patients (84.6%) in the NAFLD group had a good neurological prognosis, which was significantly lower than that of 201 patients (95.7%) in the control group (mRS score ≤ 2) (*P* = 0.005).

## Discussion

In the prospective study, 275 patients diagnosed with MMD were included. NAFLD patients and control patients had significantly different stroke-free survival rates. The NAFLD group exhibited a median survival of 19 months, which was obviously shorter than that of the control group (median survival of 23 months). MMD patients with NAFLD had a higher follow-up mRS score than controls did, indicating a worse neurological outcome (*P* = 0.005). This finding demonstrates that compared with controls, MMD patients with NAFLD had significantly poorer postoperative neurological status recovery. The findings confirm that NAFLD independently increases the risk of ischaemic stroke following surgical treatment for MMD.

NAFLD is emerging as a prominent factor of liver disease worldwide, with obesity exacerbating metabolic diseases as a global epidemic. The incidence of NAFLD has steadily increasing in recent years, and the severity of NAFLD has been confirmed to be independently associated with ischemic stroke events in previous studies [18]. The progression of NAFLD to systemic vascular diseases has always been the focus of research. According to numerous previous studies, NAFLD has indeed been established as a risk factor for cardiovascular disease [16, 24, 25]. Notably, in a 2017 cohort, NAFLD was implicated in the heightened prevalence of atherosclerotic plaques in both the carotid arteries and lower extremities [26]. Some studies have pointed out that elevated GGT levels are linked to an increased incidence of acute ischaemic stroke and myocardial infarction [19]. Elevated GGT is present in cerebrovascular, carotid and coronary artery plaques and colocalizes with foam cells. By becoming enriched in plaques, GGT maintains its enzymatic activity, induces oxidative stress, promotes the development of plaque instability and rupture, and further induces stroke or coronary heart disease [27, 28]. In the study, individuals with MMD and NAFLD exhibited significantly elevated GGT levels compared to the control group level. Consequently, MMD patients with NAFLD are more prone to postoperative ischaemic stroke events than controls are, and this association is correlated with increased GGT levels. Systemic endothelial dysfunction has been identified in individuals with NAFLD. Studies have reported a significant increase in asymmetric dimethylarginine (ADMA) among NAFLD patients, and this increase is strongly linked to cardiovascular disease. This elevation might be linked to the reduced capacity of the liver in NAFLD patients to deactivate and break down ADMA [29, 30]. Endothelial progenitor cells (EPCs) are crucial for epidermal repair in atherosclerosis. However, among NAFLD patients, there is a notable reduction in circulating EPC levels, accompanied by weakened adhesion functionality [31, 32]. This may increase the rupture and breakdown of atherosclerotic plaques, leading to vascular lumen occlusion and stroke events. Higher levels of vascular endothelial growth factor (VEGF), acting on stimulating vascular endothelial cell growth and inducing vascular proliferation, were observed in non-alcoholic steatohepatitis (NASH) patients and non-alcoholic fatty liver (NAFL) patients than in control individuals [33]. In mouse models, the use of anti-VEGFR2 treatment has been shown to enhance both steatosis and inflammation [34]. VEGF is implicated in vascular pathophysiology and actively contributing to atherosclerosis and plaque instability, thus fostering plaque formation and vulnerability and consequently elevating the risk of stroke. Moreover, increased VEGF levels significantly exacerbate the patient prognosis in patients with acute ischaemic stroke [35]. Therefore, there are complex interactions and adverse effects between NAFLD and systemic ischemic disease.

Previous studies have indicated that alterations in hepatic lipid and lipoprotein metabolism are the primary factors that increase the risk of cardiovascular disease in individuals with NAFLD [36]. Based on the baseline data, patients with NAFLD had reduced levels of HDL-C, as well as elevated levels of TG, ALT, AST, GGT and BMI than controls. The baseline characteristics of patients were consistent with those reported in previous studies [37]. Lipids, lipoproteins, and metabolites, including TG, HDL-C, and various other factors, have been shown to be significantly associated with the development of cardiovascular and cerebrovascular ischaemic events [36]. Controlling abnormal lipid metabolism, including lowering cholesterol and triglyceride levels by improving diet, increasing physical activity, and quitting smoking, can reduce the risk of stroke. Selective lipid-lowering therapy with statins is also considered necessary. In general, there is a close relationship between abnormal lipid metabolism and stroke, and maintaining healthy lipid metabolism is an important factor in preventing stroke. Taking early steps to manage abnormal lipid metabolism can reduce the incidence of stroke and maintain cardiovascular and cerebrovascular health.

NAFLD is frequently accompanied by complex multisystem diseases, making it challenging to elucidate the underlying mechanisms related to NAFLD and stroke. Lipotoxicity is recognized as one of the primary adverse outcomes associated with NAFLD, a condition characterized by the cytotoxic effects stemming [38]. Specifically, it encompasses heightened lipolysis, the release of free fatty acids, the secretion of adipokines and inflammatory cytokines, ultimately culminating in the exacerbation of insulin resistance. Notably, insulin resistance has been associated with the development of MMD [39]. Interestingly, there is also confirmed evidence that the well-established susceptibility gene Ring Finger Protein 213 (RNF213) for MMD impacts cytotoxicity and insulin resistance [40]. It has been established as a regulator of lipotoxicity induced by saturated fatty acids. However, the precise mechanism underlying this regulation warrants further investigation. NAFLD initiates with the accumulation of fat within liver cells and progresses to NASH, which is characterized by the infiltration of inflammatory cells [41]. This inflammatory process extends beyond the liver and can incite a systemic inflammatory response. This, in turn, can result in the release of various inflammatory mediators, including interleukin-6 [42], which has the potential to further exacerbate systemic inflammation [43]. Inflammation has the capacity to instigate major risk factors for stroke, such as endothelial dysfunction and atherosclerosis [44]. Furthermore, inflammation may provoke thrombosis, thereby heightening the risk of ischemic stroke [45]. According to the latest report, immune inflammation-related stimulation has emerged as an additional trigger factor for MMD, in addition to genetic factors [46]. Prior research has demonstrated the potential involvement of inflammation in MMD, suggesting a potentially more pronounced immune-inflammatory imbalance in the intermediate stages of the disease [47]. Although the precise underlying mechanism remains elusive, the double-hit theory [48, 49] of MMD emphasizes the growing significance of research on immune inflammation. In conclusion, while the precise mechanism linking NAFLD to postoperative ischemic stroke in MMD patients remains unclear, the double-hit hypothesis involving genetic factors and immune inflammation in MMD may hold the key to understanding its pathogenesis.

In summary, the prospective study of postoperative MMD patients revealed a heightened risk of ischemic stroke in individuals with MMD and NAFLD, irrespective of other stroke risk factors. Furthermore, patients with NAFLD exhibited a poorer neurological prognosis. The study suggests that reducing blood lipids and improving lipid metabolism in MMD may help prevent stroke events.

### Study strengths and limitations

This study is the first prospective cohort study of stroke events after revascularization in the setting of NAFLD, which is a strength of the study. Nevertheless, there are certain limitations in this study. Firstly, MMD is a rare disease, and despite best efforts to include patients, the size of the sample remained relatively small. This limitation may impact the robustness of the results and reduce statistical power. Secondly, the study was unable to perform liver biopsies on MMD patients, which is an important criterion for diagnosing NAFLD, to accurately determine the prevalence of NAFLD in each patient. Although ultrasonography and CT are cost-effective, safe, and accessible alternative detection methods for liver biopsy, they may reduce the reliability and accuracy of NAFLD diagnosis compared to the gold standard. Thirdly, the study did not investigate the specific causes of strokes or deaths attributed to NAFLD in patients who underwent revascularization. Fourth, the study lacks the ability to explore the impact of NAFLD on angiogenesis in MMD patients, suggesting that this mechanism warrants additional experimental validation. This requires further experimental research for a more comprehensive evaluation in the future. Fifth, in accordance with the hierarchical diagnosis and treatment system in China, patients typically initially seek emergency treatment at local municipal hospitals or community hospitals upon the onset of initial symptoms such as infarct stroke, haemorrhagic stroke, and transient ischemic attack (TIA). Following stabilization of the patient’s condition, they are subsequently transferred to our hospital for surgical treatment, and unfortunately, the study lack information regarding the acute phase of patient’s onset.

## Conclusions

NAFLD was a risk factor independent of other factors for strokes in MMD patients following revascularization, significantly impacting neurological prognosis. Preventing postoperative stroke events in MMD patients may be achieved by reducing blood lipids and correcting lipid metabolism abnormalities.

## Data Availability

The datasets used to support the findings of this study are available from the corresponding author upon reasonable request.

## Abbreviations

NAFLD: nonalcoholic fatty liver disease
MMD: moyamoya disease
MMS: moyamoya syndrome
NAFL: non-alcoholic fatty liver (also known as simple steatosis)
NASH: non-alcoholic steatohepatitis
SBP: systolic blood pressure
DBP: diastolic blood pressure
BMI: body mass index
WBC: white blood cell
LY: lymphocyte; PLT, platelet
HDL-C: high-density lipoprotein cholesterol
LDL-C: low-density lipoprotein cholesterol
Hcy: homocysteine
ALT: alanine transaminase
AST: aspartate transaminase
GGT: γ-glutamyltransferase
Cr: creatinine
UA: uric acid
TG: triglyceride
TC: total cholesterol
ApoA: apolipoprotein A
ApoB: apolipoprotein B
SD: standard deviation
IQR: interquartile range
ADMA: asymmetric dimethylarginine
NOS: nitric oxide synthase
EPCs: endothelial progenitor cells
VEGF: vascular endothelial growth factor
